# The protective effect of non-invasive low intensity pulsed electric field and fucoidan in preventing oxidative stress-induced motor neuron death via ROCK/Akt pathway

**DOI:** 10.1371/journal.pone.0214100

**Published:** 2019-03-19

**Authors:** Chih-Hsiung Hsieh, Chueh-Hsuan Lu, Yu-Yi Kuo, Guan-Bo Lin, Chih-Yu Chao

**Affiliations:** 1 Department of Physics, Lab for Medical Physics & Biomedical Engineering, National Taiwan University, Taipei, Taiwan; 2 Biomedical & Molecular Imaging Center, National Taiwan University College of Medicine, Taipei, Taiwan; 3 Institute of Applied Physics, National Taiwan University, Taipei, Taiwan; University of PECS Medical School, HUNGARY

## Abstract

With the expansion of the aged population, it is predicted that neurodegenerative diseases (NDDs) will become a major threat to public health worldwide. However, existing therapies can control the symptoms of the diseases at best, rather than offering a fundamental cure. As for the complex pathogenesis, clinical and preclinical researches have indicated that oxidative stress, a central role in neuronal degeneration, is a possible therapeutic target in the development of novel remedies. In this study, the motor neuron-like cell line NSC-34 was employed as an experimental model in probing the effects induced by the combination of non-invasive low intensity pulsed electric field (LIPEF) and fucoidan on the H_2_O_2_-induced neuron damage. It was found that single treatment of the LIPEF could protect the NSC-34 cells from oxidative stress, and the protective effect was enhanced by combining the LIPEF and fucoidan. Notably, it was observed that single treatment of the LIPEF obviously suppressed the H_2_O_2_-enhanced expression of ROCK protein and increased the phosphorylation of Akt in the H_2_O_2_-treated NSC-34 cells. Moreover, the LIPEF can be easily modified to concentrate on a specific area. Accordingly, this technique can be used as an advanced remedy for ROCK inhibition without the drawback of drug metabolism. Therefore, we suggest the LIPEF would be a promising strategy as a treatment for motor neurodegeneration and warrant further probe into its potential in treating other neuronal degenerations.

## Introduction

Amyotrophic lateral sclerosis (ALS), featuring the progressive loss of neurons similar to Alzheimer’s disease (AD) and Parkinson’s disease (PD), is a devastating and fatal neurodegenerative disease (NDD) which causes the death of motor neurons in the motor cortex, brain stem and spinal cord [1]. In a few treatments up to now [2], the progression of ALS has been reported to be somewhat slowed down, and there has yet to be a treatment which can effectively block or even halt the progressive deterioration of the disease [3]. Hence, it is imperative to develop a therapy which can effectively block or even reverse the degenerative process of neurons. To date, the etiology of ALS remains largely unknown [4], and the causes of most cases of ALS are still undefined [5]. Among the main pathogenic factors, oxidative stress has been widely reported to play a pivotal role in the pathophysiology of common NDDs [6, 7]. Apart from aging, inflammation, environmental pollutants, and nutritional factors can also induce the oxidative stress, leading to overproduction of free radical attacking neural cells [8]. It has been reported that oxidative stress could also trigger the activation of glial cells, the key factor in neuroinflammation which contributes to neurodegeneration and synaptic abnormalities [9, 10]. Besides, accumulating evidence suggests that the overproduction of reactive oxygen species (ROS) can deplete glutathione (GSH) [11] and increase the misfolded protein load in the endoplasmic reticulum (ER) [12], causing the formation of insoluble protein aggregation [13], which is a common feature for neurodegeneration. Consequently, how to prevent oxidative damage and enhance neuron regeneration could be the major therapeutic strategy in treating motor neuron degeneration.

Rho-associated protein kinase (ROCK), the downstream target protein of Rho GTPases [14], is highly expressed in neurons and different types of glial cells [15], underscoring its importance in the nervous system. It is known that ROCK acts as a central regulator in participating in a wide range of neuronal functions, such as axonal regeneration, cell cycle progression, and cell death/survival [16]. There has accumulated much evidence showing that the activation of ROCK pathway is involved in neuroinflammation and inflammation-associated oxidative stress [17, 18]. A previous study has demonstrated that the protein expression of RhoA can be directly regulated by ROS because RhoA has a redox-sensitive motif in its genetic sequence [19]; hence, it is believed that ROCK would also be activated by ROS. Besides, abnormal activation of ROCK pathway, detected in skeletal muscle of ALS patients [20], was supposed to contribute to the neuronal apoptosis [21]. Indeed, the therapeutic potency of ROCK inhibitors has been widely explored [22], showing that ROCK inhibition has beneficial effect on neuron survival [23]. In addition, ROCK inhibitors have been reported to induce favorable influences on animals, as well as the cellular models of PD [24] and AD [25, 26]. Recent and earlier studies have all suggested that the ROCK pathway could be a valuable target in the treatment of ALS and other NDDs. However, to date only two ROCK inhibitors (fasudil and ripasudil) are FDA-approved and available for clinical use [27]. They could induce intolerable side effects, such as ocular hyperemia [28, 29], abnormal hepatic function, headache, and insomnia [30]. Above all, they may be ineffective, due to insufficient concentrations passing the blood-brain barrier (BBB) [31, 32], and poor fat solubility making it difficult for them to cross the BBB via the lipid-based formulations [33]. Thus, it needs to continue looking for alternative remedies for ROCK inhibition with higher efficacy and less side effects.

Over the past decade, therapeutic electric stimulation has been demonstrated to have beneficial bioeffects in some medical applications, such as the healing of wounds and bone fracture, in addition to cancer treatment. Electrical current passing through the injury site has been shown to guide epithelial cell migration during the healing process [34], and direct current stimulation has been considered as an effective method facilitating bone-tissue formation [35]. Moreover, in vivo studies have shown that high-voltage pulsed electric current stimulation could reduce tumor size [36] and inhibit secondary tumor growth [37]. However, these treatments involve direct contact between cells and implanted electrodes, which may entail severe tissue reactions and infections, due to poor biocompatibility [38–40]. Furthermore, there are other adverse effects from this invasive modality, such as local toxicity of pH changes between anode and cathode, appearance of toxic electrode products, temperature increase, and unexpected dielectric breakdown [41–44]. Therefore, the application of low intensity non-invasive electrical stimulation is a preferred treatment. In fact, we have previously demonstrated that the non-invasive low intensity pulsed electric field (LIPEF) could significantly enhance the anticancer ability of EGCG and curcumin via the synergistic stimulation on the mitochondrial function [45, 46]. Given the successful application of LIPEF in cancer treatment, it is believed that multi-frequency components in the LIPEF signal may also induce bioeffects in neuron cells. Therefore, the non-invasive LIPEF could produce beneficial effects on neurons damaged by oxidative stress, making it a promising treatment for ALS.

On the other hand, fucoidan, a kind of sulfated polysaccharide extracted from brown seaweeds, has been extensively studied for its prominent biological activities, including anticoagulant [47], anticancer [48], immunomodulation [49], and anti-inflammation [50]. Notably, recent studies have demonstrated that fucoidan can protect against dopaminergic neuron death in vitro and in vivo models of PD [51, 52]; nevertheless, the neuroprotection was induced by high concentrations of fucoidan, and low concentration seems to be ineffective to exert any protective effect. Therefore, by means of applying other remedies to enhance the efficacy of fucoidan, the combination of fucoidan with the non-contact LIPEF stimulation could be a potential treatment for the motor neurodegeneration.

To the best of our knowledge, this is the first investigation that is conducted regarding the effect of electrical stimulations on oxidative stress related to neuron impairment. In this study, the motor neuron-like NSC-34 cell line was employed as an *in vitro* model, and the cells were exposed to non-invasive LIPEF in a non-contact manner for probing the effect of the LIPEF on the H_2_O_2_-induced neuron cell damage. The study discovered that the non-contact LIPEF treatment alone could protect the NSC-34 cells from the attack of oxidative stress via the inhibition of the H_2_O_2_-enhanced expression of ROCK protein. In addition, fucoidan was further applied to the LIPEF treatment. The results revealed that the addition of fucoidan could bring benefit effect by regulation of Bcl-2 family proteins. Therefore, the treatment combining the LIPEF and fucoidan cooperatively enhanced the neuroprotective effect. Moreover, it was observed that the combination treatment significantly inhibited the H_2_O_2_-induced neurite retraction. The study, therefore, proves for the first time that the non-contact LIPEF induces protective effect in the motor neuron-like cells, which may shed light on the treatment of other NDDs.

## Materials and methods

### Cell culture

The mouse motor neuron-like cell line NSC-34 was obtained from Cellutions Biosystems Inc. (Toronto, ON, Canada). NSC-34 cells were maintained in Dulbecco’s modified Eagle’s medium (DMEM) (HyClone, South Logan, UT, USA) supplemented with 10% fetal bovine serum (FBS) (HyClone), 100 unit/ml penicillin (Gibco Life Technologies, Grand Island, NY, USA), and 100 mg/ml streptomycin (Gibco Life Technologies) in a humidified 5% CO_2_ incubator at 37°C. Cells were harvested with 0.05% trypsin–0.5 mM EDTA solution (Gibco Life Technologies) and prepared for in vitro experiments when cells approached about 80% confluence. Before each treatment, NSC-34 cells were differentiated in 10 μM retinoic acid (Sigma-Aldrich, St. Louis, MO, USA) for 7 days.

### Experimental setup for exposure of the cells to non-contact LIPEF

The LIPEF device, which we had previously described [45, 46, 53], was used for exposure of the NSC-34 cells to various strength of electric field. The cells were placed between two copper flat and parallel electrodes. Due to the existence of air gap between the electrodes and culture medium [45, 46, 53], our device provided a non-contact method for the application of non-invasive LIPEF. Consecutive pulses with different electric field intensities (15, 30, 60, and 90 V/cm), pulse duration 2 ms, and the frequency 2 Hz were applied across the electrodes. Cells treated with continuous exposure of the non-contact LIPEF were kept at 37°C in a humidified atmosphere of 5% CO_2_.

### Fucoidan treatment

Fucoidan has been reported to exhibit anti-inflammatory and anti-oxidative properties [54, 55], and therefore it has the potential to be applied to the treatment of motor neurodegeneration. In this study, the fucoidan (from *Fucus vesiculosus*) was purchased from Sigma-Aldrich and dissolved in distilled water as a stock solution. NSC-34 cells were seeded in 35 mm diameter cell petri dishes at the cell density about 1 × 10^5^ cells/dish. Subsequently, cells were treated with various concentrations of fucoidan (0, 10, 20, 50, 100, 150, and 200 μg/ml). After cell viability assay for optimal concentration determination, 100 μg/ml fucoidan was employed in the following experiments.

### Cell viability assay

3-(4,5-dimethylthiazol-2-yl)-2,5-diphenyltetrazolium bromide (MTT) (Sigma-Aldrich) was dissolved in distilled water to make a 5 mg/ml stock solution. Cells were pretreated with LIPEF, fucoidan, or in combination for 1 h. Subsequently, H_2_O_2_ was further added to the culture medium. Cell viability was measured at 24 h after the treatment of H_2_O_2_. The medium was replaced with fresh one containing 0.5% mg/ml MTT for 2 h incubation at 37°C. Subsequently, the supernatant was removed, and then 1 ml DMSO was added to dissolve the formazan crystals. The absorbance was measured at 570 nm using Multiskan GO spectrophotometer (Thermo Scientific, NH, USA).

### Reactive oxygen species (ROS) analysis by flow cytometry

After the treatment of H_2_O_2_ for 24 h, NSC-34 cells were harvested and washed twice with PBS. Then, the cells were resuspended in PBS and incubated with 10 μM 2’,7’-dichlorofluorescin diacetate (DCFH-DA) (Sigma-Aldrich) for 30 min at 37°C in the dark. After interaction with ROS, the non-fluorescent DCFH-DA was converted to the fluorescent 2',7'-dichlorofluorescein (DCF) [56]. The fluorescence intensity was immediately measured using flow cytometry. Statistical analysis of fluorescence was recorded by FACSCanto II system (BD Biosciences, San Jose, CA, USA).

### GSH colorimetric assay

For protecting cells from the damage caused by ROS, cellular GSH is oxidized to glutathione disulfide (GSSG). Hence, the cellular GSH/GSSG ratio is a useful indicator of oxidative stress [57], and it was measured using a GSH colorimetric detection kit (BioVision Inc., Milpitas, CA, USA). After the treatment of H_2_O_2_ for 24 h, NSC-34 cells were harvested and washed twice with PBS. Then, the cells were processed according to the manufacturer’s instructions. The absorbance was measured at 412 nm using Multiskan GO spectrophotometer (Thermo Scientific).

### Mitochondrial membrane potential (MMP) detection

The detection of MMP was carried out using 3,3-dihexyloxacarbocyanine iodide (DiOC_6_(3)) (Enzo Life Sciences International Inc., NY, USA) [58]. After the treatment of H_2_O_2_ for 24 h, NSC-34 cells were collected and incubated with PBS containing 20 nM DiOC_6_(3) at 37°C for 30 min in the dark. Subsequently, the fluorescence intensity was immediately measured using flow cytometry. Statistical analysis of fluorescence was recorded by FACSCanto II system (BD Biosciences).

### BiP ELISA (enzyme-linked immunosorbent assay)

After the treatment of H_2_O_2_ for 24 h, cells were washed with PBS and then subjected to freeze-thaw cycles for cell lysis. The lysates were then centrifuged, and the supernatant was collected and used for the determination of protein concentration by BSA method. Equal amounts of protein extract were loaded to a commercial BiP ELISA kit (MyBioSource, San Diego, CA, USA) and processed according to the manufacturer’s instructions. The absorbance was measured at 450 nm using Multiskan GO spectrophotometer (Thermo Scientific).

### Fluorescent labeling

Cells were grown on sterile glass coverslips, treated with different conditions, and then washed twice with PBS. For the observation of neurite outgrowth, the cells were fixed in 4% paraformaldehyde (PFA) (Sigma-Aldrich) for 15 min and then permeabilized with 0.1% Triton X-100 (Bioshop Canada Inc., Burlington, Ontario, Canada) in PBS for 15 min at 37°C. Then, 1% bovine serum albumin (BSA) was used for blocking the non-specific protein binding. The cells were then incubated overnight with primary anti-beta-III tubulin (1:200 dilution; Gentex, Irvine, CA, USA) at 4°C. After being rinsed with PBS, NSC-34 cells were incubated with a secondary Alexa Fluor 647-conjugated antibody (1:1000 dilution; Abcam, Cambridge, MA, USA). Finally, coverslips were mounted in 10 μl glycerol-based mounting medium.

For the observation of nuclear morphology, the cells were fixed in 4% PFA (Sigma-Aldrich) for 15 min, and then the coverslips were directly mounted in 10 μl mounting medium containing DAPI (Abcam). All the fluorescent images were observed and recorded using Zeiss Axio Imager A1 microscope.

### Western blotting analysis

After the treatment of H_2_O_2_ for 24 h, cells were washed with PBS and then lysed on ice for 30 m in lysis buffer (50 mM Tris-HCl, pH 7.4, 0.15 M NaCl, 0.25% deoxycholic acid, 1% NP-40, 1% Triton X-100, 0.1% SDS, 1 mM EDTA) containing fresh protease and phosphatase inhibitor cocktail (Millipore, Billerica, MA, USA). The lysates were then centrifuged, and the supernatant was collected and used for the determination of protein concentration by BSA method. Equal amounts of protein extract were loaded in the 12% SDS-PAGE wells and transferred to polyvinylidene difluoride (PVDF) membranes (Millipore). The membranes were blocked with TBST washing buffer (20 mM Tris, 150 mM NaCl, and 0.1% Tween 20) containing 50 g/L nonfat milk powder for 1 h and incubated overnight with primary antibodies at 4°C, followed by three rinses with TBST washing buffer. Then, the membranes were incubated with horseradish peroxidase-coupled secondary antibodies for 1 h at room temperature. In this study, primary antibodies were purchased from the following: anti-GAPDH, anti-total Akt, and anti-Bcl-2 (Gentex); anti-Bax (Santa Cruz Biotechnology, Santa Cruz, CA, USA); anti-phospho-Akt (Ser473) (Cell Signaling Technology, Danvers, MA, USA); anti-ROCK (Abcam). The secondary antibodies were purchased from Jackson ImmunoResearch Laboratories (West Grove, PA, USA). All the antibodies were diluted at the optimal concentration according to the manufacturer’s instructions. Finally, protein bands were detected using chemiluminescence ECL kit (T-Pro Biotechnologies, New Taipei City, Taiwan). For normalization of p-Akt, total Akt was used as an internal control; for normalization of other proteins, GAPDH was used as an internal control.

### Neuron morphology quantification

Neuron morphology features, such as soma number, neurite length, and neurite branching complexity, were analyzed using NeurphologyJ [59], which is an ImageJ plugin for automatic quantification of neuronal morphologies. Beta-III tubulin-stained images were used as the original input of NeurphologyJ.

### Statistical analysis

Each experiment was repeated three times, and statistical analysis was conducted using SigmaPlot version 12.5 for Windows (Systat Software, Inc., San Jose, CA, USA). Student’s t-test was used to compare two groups. One-way analysis of variance (ANOVA) was employed to compare multiple groups with Turkey’s test as post hoc test. The test performed for each experiment is indicated in the figures, and the results with P < 0.05 were considered statistically significant. In the figures, * is used for P < 0.05, ** for P < 0.01, and *** for P < 0.001.

## Results

### Effect of the non-invasive LIPEF and fucoidan on the H_2_O_2_-induced cell death in NSC-34 cells

**Fig 1:**
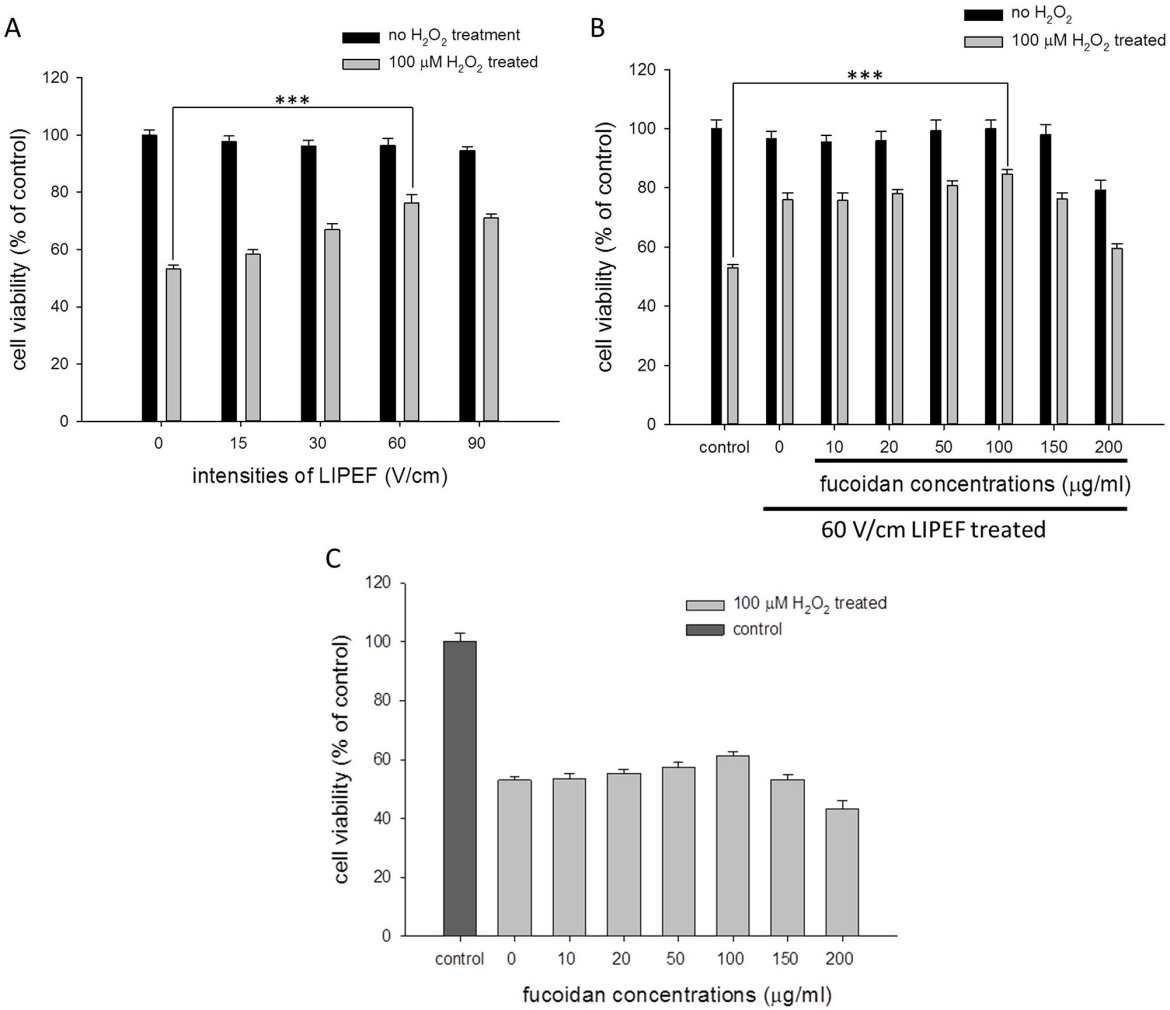
Effect of the combination treatment of the LIPEF and fucoidan on the cell viability of H_2_O_2_-treated NSC-34 cells. The cell viability was determined using MTT assay at 24 h after the treatment of 100 μM H_2_O_2_. (A) NSC-34 cells were pretreated with different intensities of LIPEF for 1 h and then exposed to 100 μM H_2_O_2_ in the continuous administration of the LIPEF for another 24 h. (n = 3, Student’s t-test; *** is used for P < 0.001). (B) Different concentrations of fucoidan were combined with the 60 V/cm LIPEF treatment on the 100 μM H_2_O_2_-treated NSC-34 cells. (n = 3, Student’s t-test; *** is used for P < 0.001). (C) Fucoidan alone at various concentrations (0 to 200 μg/ml) showed little protective effect.

In order to understand whether the non-contact LIPEF alone or together with fucoidan could attenuate the H_2_O_2_-induced neuron cell damage, the NSC-34 cells were pretreated with the LIPEF alone or in combination with fucoidan for 1 h, and the cells were then exposed to 100 μM H_2_O_2_ in the continuous administration of the LIPEF alone or in combination with fucoidan for another 24 h. As shown in Fig 1A, the cell viability of NSC-34 cells exposed to 100 μM H_2_O_2_ for 24 h without the LIPEF treatment was reduced to 53% of the control value, and it was rescued and increased to 58%, 67%, 76%, and 71% of the control value when the cells were treated with 15, 30, 60, and 90 V/cm of the LIPEF, respectively. Then, we further applied various concentrations of fucoidan (0 to 200 μg/ml) to the 60 V/cm LIPEF treatment, and the 100 μg/ml fucoidan showed the optimal enhancement on the LIPEF-induced protective effect with the cell viability larger than 85% of the control value, as shown in Fig 1B. Notably, fucoidan alone at these concentrations exhibited little protective influence on the H_2_O_2_-induced impairment (Fig 1C). The results revealed that the LIPEF treatment alone could protect the NSC-34 cells from the oxidative stress, and the combination of the LIPEF and fucoidan further enhanced the protective effect, although fucoidan alone had an insignificant defensive effect. Based on these results, the following experiments were carried out using 60 V/cm LIPEF and 100 μg/ml fucoidan.

### Effects of the LIPEF and fucoidan on the intracellular ROS and the GSH/GSSG ratio of NSC-34 cells

**Fig 2:**
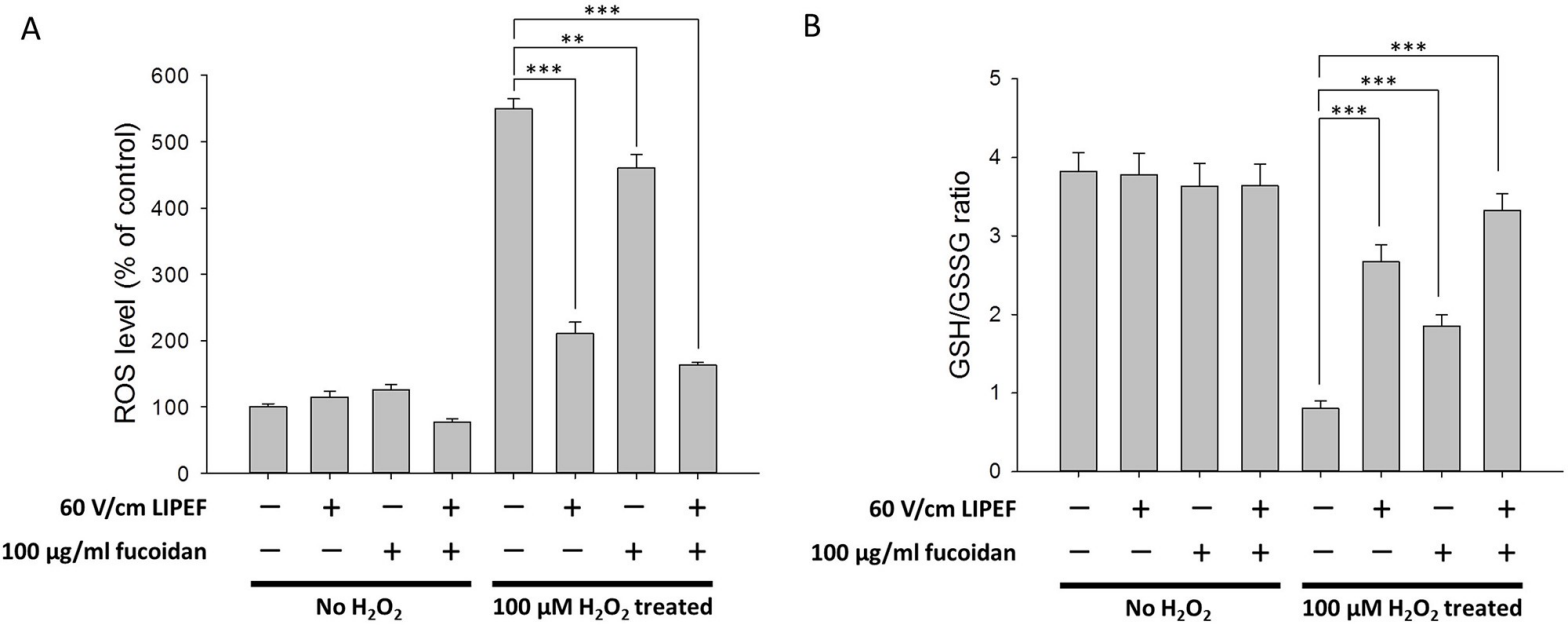
The protective effects on the ROS level and the GSH/GSSG ratio in the H_2_O_2_-treated NSC-34 cells. (A) The ROS level was measured using DCFH-DA assay. (n = 3, one-way ANOVA, Turkey’s test; ** is used for P < 0.01 and *** for P < 0.001). (B) The GSH/GSSG ratio was determined using GSH colorimetric assay. (n = 3, Student’s t-test; *** is used for P < 0.001). These results showed that the protective effects of the LIPEF and fucoidan could reduce the oxidative stress in the NSC-34 cells exposed to H_2_O_2_ for 24 h.

We further investigated whether the intracellular ROS production could be decreased when the NSC-34 cells were treated with the combination treatment, so the DCFH-DA assay [60] was employed to analyze the ROS levels. As shown in Fig 2A, the DCF fluorescence was significantly increased up to 550% of the control value when the cells were exposed to 100 μM H_2_O_2_ for 24 h without the treatment of LIPEF or fucoidan. We found the ROS levels were obviously reduced to 210% and 460% of the control value when cells challenged with H_2_O_2_ were single treated with 60 V/cm LIPEF and 100 μg/ml fucoidan, respectively. Moreover, the combination treatment of the LIPEF and fucoidan dramatically decreased the H_2_O_2_-elevated ROS level to 163% of control value. In addition, since the disorder of GSH metabolism is a major feature of ongoing oxidative stress in motor neurodegeneration [61], the GSH/GSSG ratio of NSC-34 cells was also measured. As shown in Fig 2B, the GSH/GSSG ratio was significantly decreased when the cells were challenged with H_2_O_2_ for 24 h without the treatment of LIPEF or fucoidan. Notably, the combination treatment of the LIPEF and fucoidan could prevent or attenuate GSH depletion in the H_2_O_2_-treated NSC-34 cells. Collectively, these results revealed that the protective effect of this combination treatment could reduce the oxidative stress in the NSC-34 cells exposed to H_2_O_2_.

### Effects of the LIPEF and fucoidan on the MMP, ER stress, and nuclear morphology of NSC-34 cells

**Fig 3:**
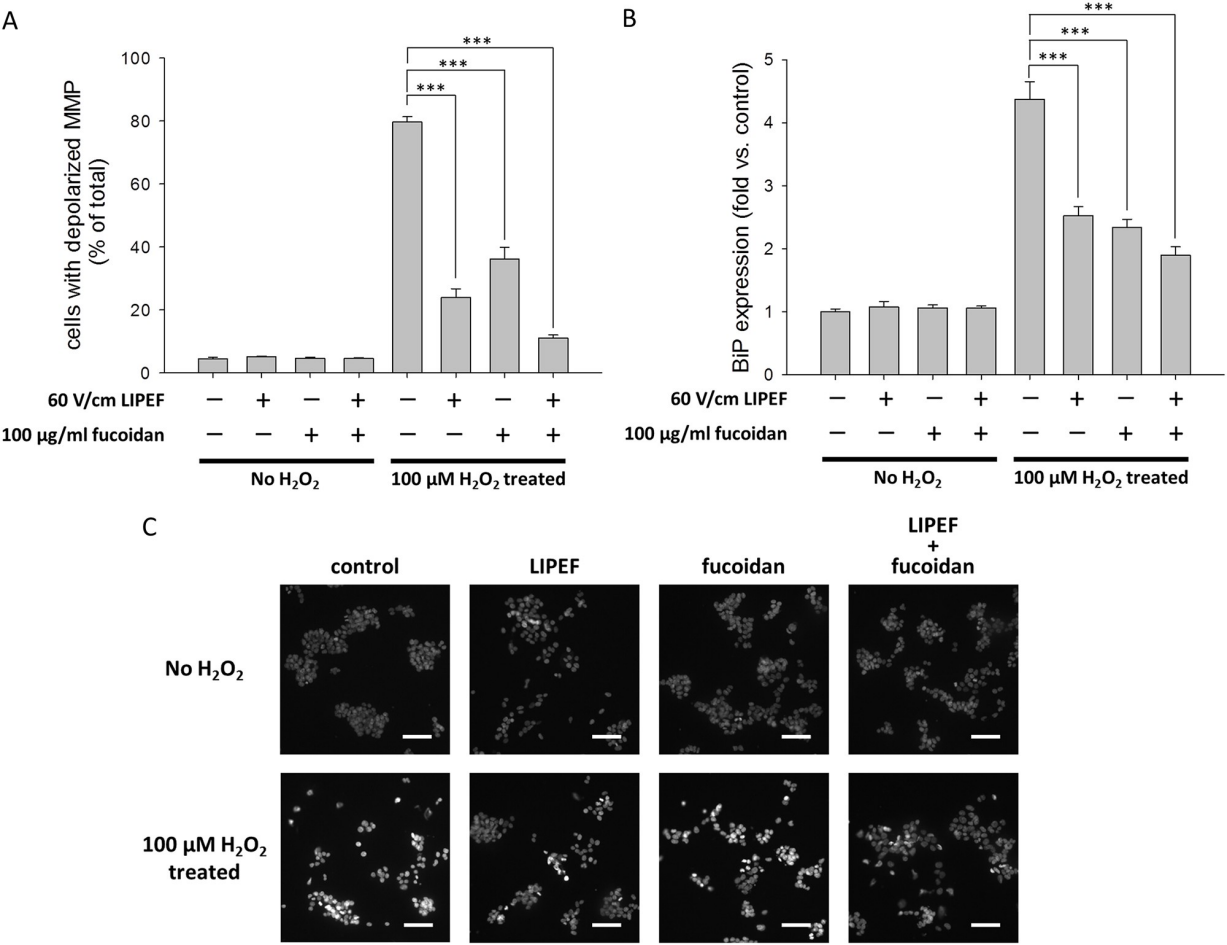
The protective effects on the MMP, the ER stress, and the nuclear condensation in the H_2_O_2_-treated NSC-34 cells. (A) The dissipation of MMP was determined using DiOC_6_(3) staining. (n = 3, one-way ANOVA, Turkey’s test; *** is used for P < 0.001). (B) The ER stress was studied by ELISA method to measure the protein expression of BiP. (n = 3, one-way ANOVA, Turkey’s test; *** is used for P < 0.001). (C) The nuclei morphology was analyzed using DAPI staining under Zeiss Axio Imager A1 microscope. Scale bar = 20 μm. These results revealed that the protective effects of the LIPEF and fucoidan could maintain the mitochondrial function, reduce the ER stress, and suppress the apoptotic response in the NSC-34 cells exposed to H_2_O_2_ for 24 h.

Following, we used DiOC_6_(3) [58] to detect the MMP so as to better understand the relationship between the MMP and the protective effect induced by the LIPEF alone or in combination with fucoidan. As shown in Fig 3A, compared with 4.5% in the control cells, the percentage of cells with depolarized MMP drastically increased up to 79.7% after the exposure to 100 μM H_2_O_2_ for 24 h without the treatment of LIPEF or fucoidan. We found that the LIPEF alone could attenuate the H_2_O_2_-induced dissipation of MMP. Moreover, the combination treatment of the LIPEF and fucoidan noticeably suppressed the H_2_O_2_-induced dissipation of MMP, and the percentage of cells with depolarized MMP was further reduced to 11.2%. Next, we examined whether the ER stress of cells was also influenced by the combination treatment of the LIPEF and fucoidan. Hence, we measured the protein expression of BiP, because BiP is the major Ca^2+^ homeostasis-related chaperone to control the activity of transmembrane ER stress sensors [62]. As shown in Fig 3B, the expression of BiP was drastically decreased after the exposure of NSC-34 cells to 100 μM H_2_O_2_ for 24 h without the treatment of LIPEF or fucoidan. It was observed that the single treatment of the LIPEF or fucoidan could reduce the H_2_O_2_-induced increase in the expression of BiP. In addition, the combination treatment further enhanced the reduction of BiP expression in the H_2_O_2_-treated cells. Moreover, we utilized DAPI staining to investigate the protective effect on the nuclear morphology of NSC-34 cells. In Fig 3C, it was found that the exposure to 100 μM H_2_O_2_ for 24 h significantly induced nuclear chromatin condensation in NSC-34 cells, and each single treatment of the LIPEF or fucoidan could reduce the H_2_O_2_-induced nuclear chromatin condensation. Additionally, the combination treatment of the LIPEF and fucoidan practically prevented the H_2_O_2_-induced nuclear chromatin condensation (see Fig 3C). The result indicated that the protective effect of this combination treatment could suppress the apoptotic response in the NSC-34 cells challenged with ROS stress.

### Role of the ROCK and Akt pathways in the neuroprotective effect of the LIPEF and fucoidan

**Fig 4:**
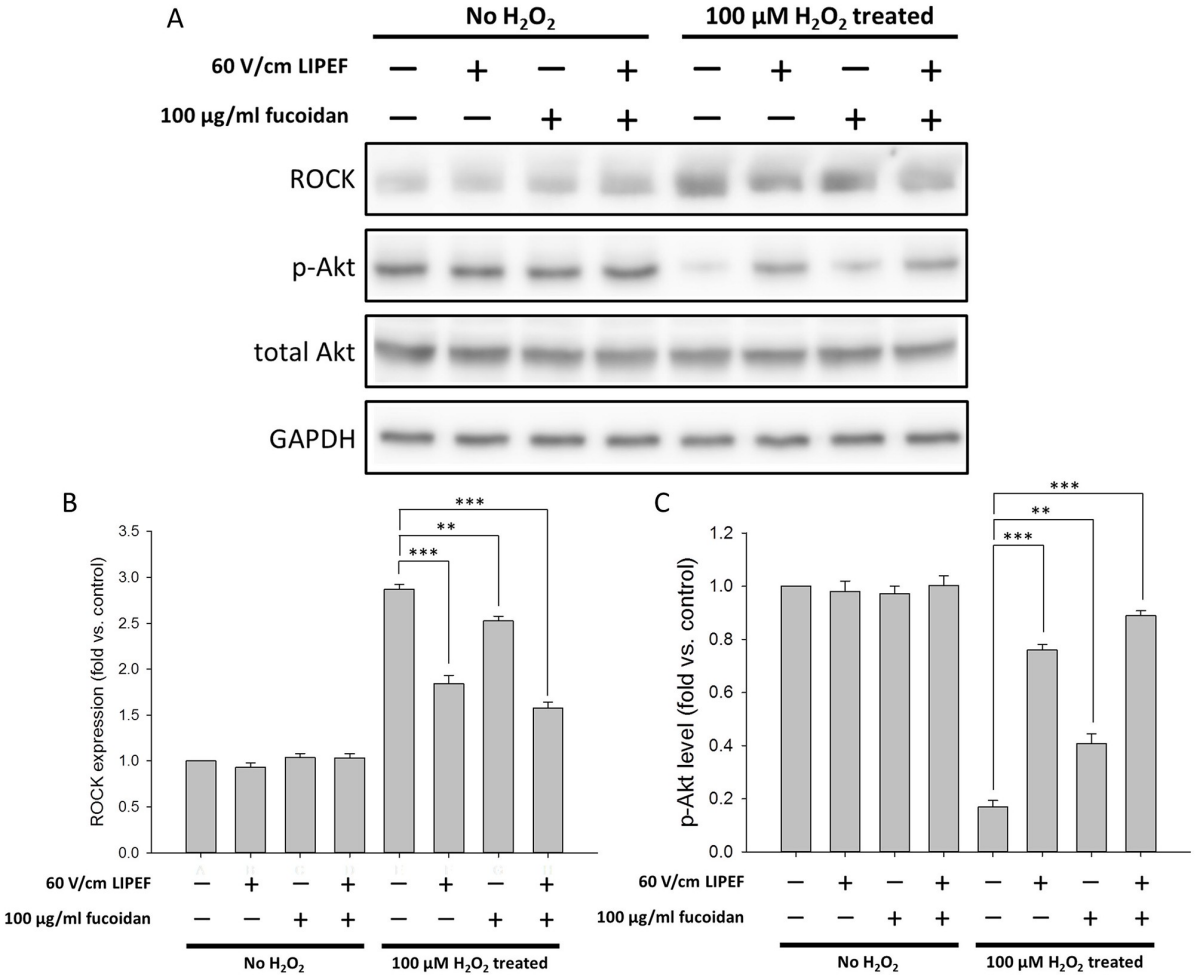
Effects of the combination treatment on the protein levels of ROCK and p-Akt. (A) Protein expression levels of ROCK and p-Akt were measured using Western blot analysis. The expression level of ROCK and p-Akt were normalized to GAPDH and total Akt, respectively. Each relative expression level was compared with control. (B) ROCK band intensities and (C) p-Akt band intensities were quantified to understand the mechanism underlying the protective effect. (n = 3, Student’s t-test; ** is used for P < 0.01 and *** for P < 0.001).

ROCK pathway is known to be involved in a wide range of fundamental cellular functions, and ROCK inhibitors have been reported to reduce the oxidative injury in various cells through the Akt survival pathway [63]. Consequently, to further identify whether the protective effect of the LIPEF and fucoidan was related to the ROCK pathway, we examined the protein expression of ROCK in the cells that were pretreated with the LIPEF and fucoidan for 1 h prior to the treatment of H_2_O_2_ for 24 h. As shown in Fig 4A, the expression of ROCK was significantly increased in the cells exposed to 100 μM H_2_O_2_ for 24 h without the treatment of LIPEF or fucoidan. In Fig 4B, we found that fucoidan slightly reduced the H_2_O_2_-induced increase in the expression of ROCK. In contrast, both of the single treatment of 60 V/cm LIPEF and the combination treatment of the LIPEF and fucoidan could significantly reduce the H_2_O_2_-induced increase in the expressions of ROCK. This result revealed that the neuroprotection induced by the LIPEF would be related to the suppression of the H_2_O_2_-elevated expression of ROCK. Following, we measured the phosphorylation of Akt to evaluate whether the Akt was also involved in the protective effect. As shown in Fig 4C, in comparison to the untreated control cells, the level of phosphorylated Akt (p-Akt) was drastically decreased in the cells exposed to 100 μM H_2_O_2_ for 24 h without the treatment of LIPEF or fucoidan. Besides, it was observed that the single treatment of fucoidan had little effect on the H_2_O_2_-induced decrease in the phosphorylation of Akt. However, we found that both of the single treatment of the LIPEF and the combination treatment of the LIPEF and fucoidan could induce an obvious recovery of the phosphorylation of Akt. This result suggested that the LIPEF could give the neuron cells a physical cue to trigger the protection signal through the ROCK pathway, and further the Akt signaling was reactivated for the promotion of cell survival.

### Relationship between the protective effect and the Bcl-2 family proteins

**Fig 5:**
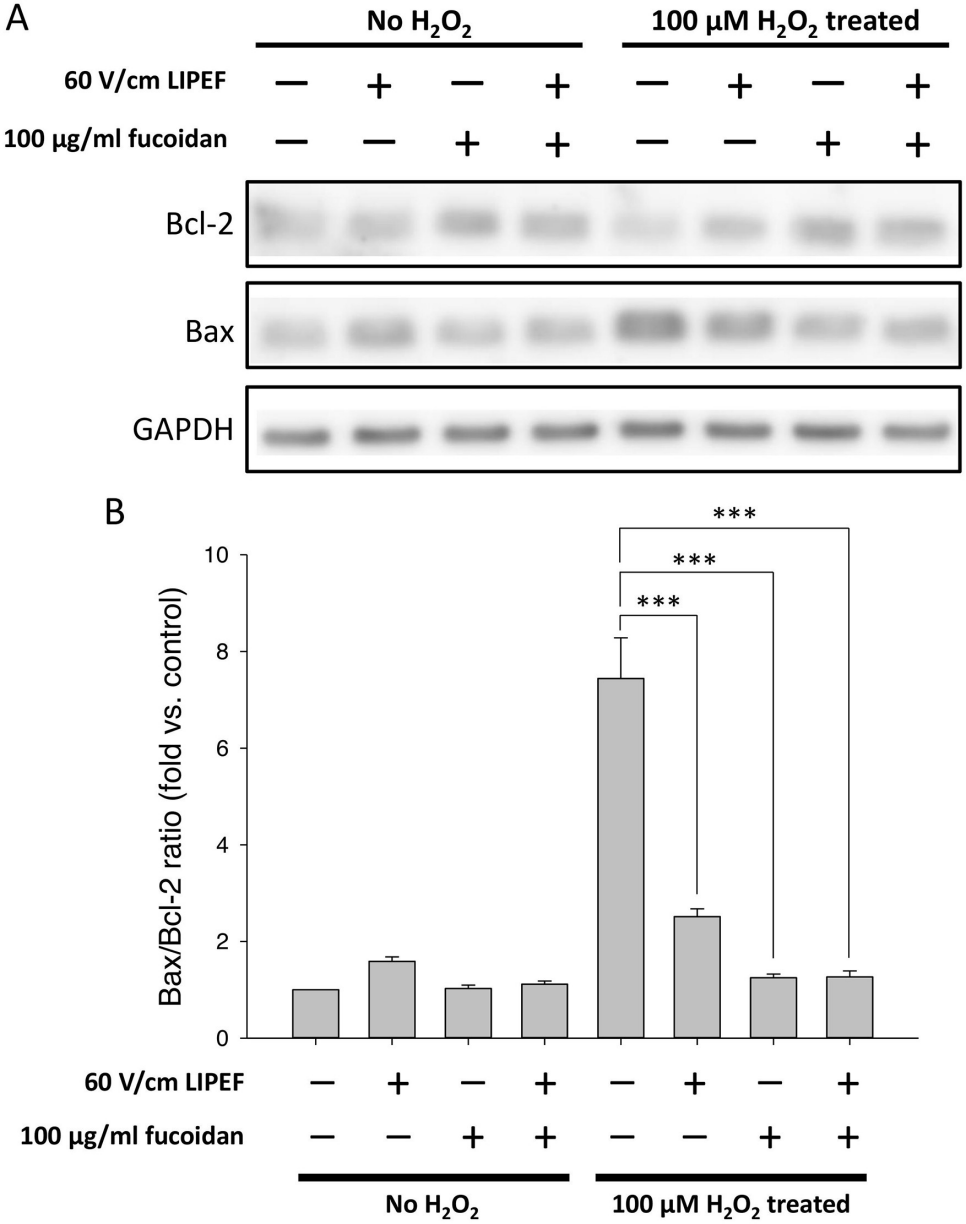
Effects of the combination treatment on the protein levels of Bcl-2 and Bax. (A) Protein expression levels of Bcl-2 and Bax were measured using Western blot analysis. Each relative expression level was normalized to GAPDH and compared with control. (B) Band intensities were quantified to obtain the Bax/Bcl-2 ratio. (n = 3, Student’s t-test; *** is used for P < 0.001).

Since the Bcl-2 family proteins serve as the regulators of cell death under ROS stress, we analyzed the expression levels of Bcl-2 and Bax to check whether the protective effect was associated with the expression regulation of the Bcl-2 family proteins. As shown in Fig 5A and 5B, when NSC-34 cells were exposed to 100 μM H_2_O_2_ for 24 h without the treatment of LIPEF and fucoidan, the expression levels of Bcl-2 and Bax were dramatically decreased and increased, respectively, leading to a significant elevation of the Bax/Bcl-2 ratio. Interestingly, we found that the combination treatment of the LIPEF and fucoidan significantly up-regulated the expression of Bcl-2 and down-regulated the expression of Bax in the cells challenged with H_2_O_2_. It is noteworthy that the H_2_O_2_-elevated Bax/Bcl-2 ratio was more reduced in the single fucoidan treatment than in the single LIPEF treatment, suggesting that the role of fucoidan in the combination treatment could regulate the expression of the Bcl-2 family proteins for the protection effect. Consequently, the combination of the LIPEF and fucoidan cooperatively promoted the cell survival against oxidative stress.

### The neuroprotective effect of the LIPEF and fucoidan on the H_2_O_2_-induced neurite retraction

**Fig 6:**
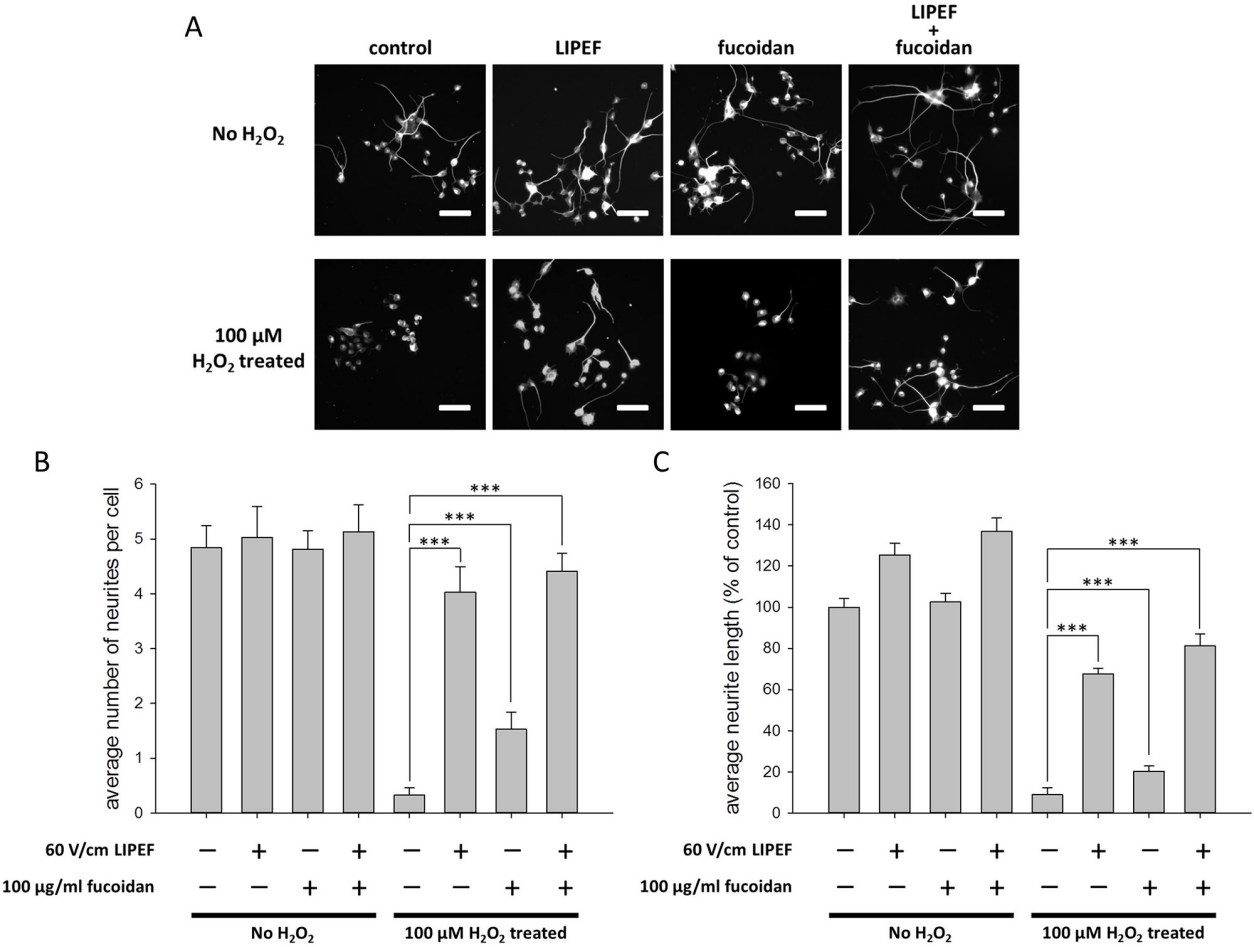
Effect of the combination treatment on the H_2_O_2_-induced neurite retraction. (A) The morphology of neuron cells was observed using immunofluorescence staining of beta-III tubulin under Zeiss Axio Imager A1 microscope. Scale bar = 20 μm. The average number of neurites per cell (B) and the average neurite length (C) were quantified to evaluate the neuroprotective effect. (n = 8, one-way ANOVA, Turkey’s test; *** is used for P < 0.001).

Since any protective strategy adopted in preserving neural tissue is not sure to prevent spontaneous degeneration of neurite, we further examined the effect of the LIPEF combined with fucoidan on the neurite retraction induced by 100 μM H_2_O_2_. Here, we employed the immunofluorescence observation using beta-III tubulin staining to study the distribution and the morphology of neurons. As shown in Fig 6A, it was obviously observed that the average neurite length and the average number of neurites per cell were sharply decreased after NSC-34 cells were exposed to 100 μM H_2_O_2_ for 24 h without the treatment of LIPEF or fucoidan. In addition, we found that the single fucoidan treatment faintly impeded the reduction in neurite lengths and numbers. In contrast, as shown in Fig 6B and 6C, both of the single LIPEF treatment and the combination treatment of the LIPEF and fucoidan significantly prevented the H_2_O_2_-induced neurite degeneration, and the average neurite length as well as the average number of neurites per cell were only slightly reduced in comparison with those of the untreated control (Fig 6B and 6C). The result confirms that the non-contact LIPEF treatment alone could not only promote neuronal survival but also prevent the neurite degeneration when NSC-34 cells were challenged with the oxidative stress caused by H_2_O_2_. Moreover, the study shows that the combination treatment using the non-contact LIPEF and fucoidan could further enhance these neuroprotective effects. Collectively, our results show the potential application of the non-contact LIPEF combined with fucoidan in preventing the oxidative stress-induced neurite degeneration and neuron death.

## Discussion

In this study, we have evaluated the neuroprotective effect of the LIPEF and the fucoidan on the H_2_O_2_-treated NSC-34 cells. NSC-34 cells are often employed in studying the pathogenesis of ALS in vitro, due to the similarity of their morphological and physiological properties to motor neurons [64]. Indeed, several studies [65–67] have also published the research findings by employing NSC-34 cells exposed to H_2_O_2_ for building up the in vitro model of motor neurodegeneration. Progressive degeneration of the motor neurons in ALS leads to the loss of muscle control before paralysis and then death [68]. To date, a growing body of evidences has indicated that oxidative stress, ER stress, and mitochondrial dysfunction are highly related to the pathogenesis of common NDDs, such as ALS, AD, and PD [69, 70], and oxidative stress has been indicated to play the central role in the pathophysiology of these diseases [6, 7]. Therefore, the development of antioxidative treatments that relieve ROS stress and enhance neuron survival in the early stage of neurodegeneration is a promising approach for preventing cell death and even improving neural repair [71].

Here, our study finds that the LIPEF alone could significantly attenuate the ROS level in the H_2_O_2_-treated NSC-34 cells and further reduce the H_2_O_2_-induced neuronal cell death. In addition, the combination of the LIPEF and the fucoidan could further enhance the neuroprotective effect, with the cell viability being restored from 53% to 85% of the control value. Also, the study shows that the single treatment of the LIPEF maintained the MMP and suppressed the H_2_O_2_-induced expression of ROCK in the NSC-34 cells. Moreover, the LIPEF increased the phosphorylation of Akt, which could lead to higher survival responses. Meanwhile, further application of fucoidan induced anti-apoptotic effect via the up-regulation of Bcl-2 expression and the down-regulation of Bax expression. Thus, the treatment combining the LIPEF and fucoidan cooperatively affected the intracellular signaling pathways and elevated the neuroprotective effect in defending the NSC-34 cells against the damage of ROS stress. Furthermore, the neuronal morphology almost remained intact once the cells were treated with the LIPEF to endure the H_2_O_2_ exposure, underscoring the ability of this non-contact LIPEF in preventing the ROS-induced neurite retraction and retaining the neuronal structural integrity.

In fact, the peculiar activation of ROCK signaling has been reported to induce and result in the neuronal apoptosis in various models of neurodegeneration [20, 72–74]. Besides, several studies have demonstrated that the treatment of ROCK inhibitors could elicit positive effects on neurodegeneration [75–78] and reactivate Akt kinase for cell survival [21, 24]. Notably, the protein expression of RhoA has been shown to be directly regulated by ROS because of the redox-sensitive motif in its genetic sequence [19]; thus, it is suggested that ROCK can also be activated by the ROS-induced activation of RhoA. In this study, it was discovered that the treatment of the LIPEF alone could markedly reverse the H_2_O_2_-induced increase in the expression of ROCK and thus reduce the H_2_O_2_-induced cell death of NSC-34 cells. It is suggested, therefore, that the LIPEF treatment may play a role similar to the ROCK inhibitors in alleviating the ROS stress and strengthening the survival signaling pathways, as evidenced by the upturn in phosphorylation of Akt. Meanwhile, the administration of the fucoidan lessened the H_2_O_2_-induced increase in the Bax/Bcl-2 ratio, underscoring the beneficial effect of fucoidan in enhancing the LIPEF-induced neuroprotection. The study, therefore, shows that the treatment combining the LIPEF and fucoidan could cooperatively maintain MMP, reduce the ER stress, avoid nuclear condensation, and increase the cell viability at a time when the NSC-34 cells were exposed to H_2_O_2_.

Furthermore, different from the previous method employing high intensity AC signal stimulation, our non-contact LIPEF technique can be seen as the sum of many sinusoidal subcomponents with multiple frequencies, which can simultaneously interact with molecules, proteins, and cells over the experimental period of time. The pulse frequency and the field intensity of our non-contact LIPEF are very important factors for biological applications. It is suggested, therefore, to optimize the LIPEF treatment via adjusting parameters when dealing with different types of cells. Also, the parameters of the LIPEF could vary with a different agent in another combination treatment. In this work, the finding of the study suggests a possible relationship between the LIPEF signal and inherent frequencies of the NSC-34 cells which induce the neuroprotective effect, making the LIPEF a possible novel treatment for neurodegeneration.

As far as we know, this is the first study confirming that the LIPEF alone could induce neuroprotective effect against the H_2_O_2_-induced cell damage and its finding shows the underlying mechanism might be associated with the regulation of ROCK signaling pathway. In our study, the non-invasive LIPEF instrument delivered stimulations in a non-contact manner, greatly facilitating the development of clinical therapy. Since there was a dielectric air gap separating the electrodes from the cells, it could avoid the harmful effects of direct contact, such as the toxic electrode products and undesirable leakage current in the body, as mentioned previously [42, 44]. The method of the study is safer for application to patients with degenerative brain diseases. Besides, the electric field with sufficient penetration could be modified for concentration on a specific area, enabling continuous application of the LIPEF stimulation to the treatment site for longstanding exposure, which could prevent BBB problem in drug delivery. In other words, the LIPEF could serve as an approach for the ROCK inhibition without the limitation of drug metabolism, and it should be more convenient and effective for the medical application than the ROCK-inhibitor drugs. Given close association of ROCK signaling pathway to the pathomechanism of many neurodegenerative disorders [20, 73, 74, 79], the application of the non-invasive LIPEF in other NDDs may also produce neuroprotective effects. The study, therefore, could become an inspiring precedent for future exploration for optimal LIPEF conditions for the treatment of neurodegeneration.

In summary, the study demonstrates for the first time that the LIPEF alone or in combination with fucoidan could significantly decrease the ROS level in the NSC-34 cells and lessen the cell death. In particular, the neuroprotective effect was found to be associated with the inhibition of the H_2_O_2_-induced activation of ROCK pathway. Moreover, it was observed that application of the LIPEF to the cells could significantly inhibit the H_2_O_2_-induced neurite retraction. The findings of the study show that the non-contact LIPEF treatment could avoid the disadvantages of the invasive method, function as a remedy for the ROCK inhibition, and prevent the H_2_O_2_-induced neurite degeneration, underscoring its potential to block or retard the degeneration of neurons for application in treatment. Consequently, it is worthwhile to conduct further research on the application of the non-invasive LIPEF technique in therapeutic treatment for current and future patients suffering ALS or other NDDs.

## Supporting information

S1 File Raw data of MTT assay. (RAR)

S2 File Raw data of ROS and GSH assay. (RAR)

S3 File Raw data of MMP and BiP ELISA assay. (RAR)

S4 File Raw image of DAPI staining. (RAR)

S5 File Raw data of ROCK and p-Akt. (RAR)

S6 File Raw data of Bcl-2 and Bax. (RAR)

S7 File Raw image of neurite. (RAR)

S8 File Raw data of neurite number and length. (RAR)
